# Impact of NaH on the Electrochemical Performance of Sodium Batteries

**DOI:** 10.1021/acsomega.4c08310

**Published:** 2025-01-14

**Authors:** Alexander Thomas, Björn Pohle, Marcus Schmidt, Henrik-Gerd Bischoff, Marius Lau, Felix Heubner, Stefan Kaskel, Daria Mikhailova

**Affiliations:** † Leibniz Institute for Solid State and Materials Research (IFW) Dresden e. V., Helmholtzstraße 20, 01069 Dresden, Germany; ‡ Chemische Metallkunde, Max-Planck-Institut für Chemische Physik fester Stoffe, Nöthnitzer Straße 40, 01187 Dresden, Germany; § Schaufler-Professur für Kaelte-, Kryo- und Kompressorentechnik, Technische Universität Dresden, 01062 Dresden, Germany; ∥ Fraunhofer Institute for Manufacturing Technology and Advanced Materials IFAM, Winterbergstraße 28, 01277 Dresden, Germany; ⊥ Department of Inorganic Chemistry, Technische Universität Dresden, Bergstraße 66, 01069 Dresden, Germany

## Abstract

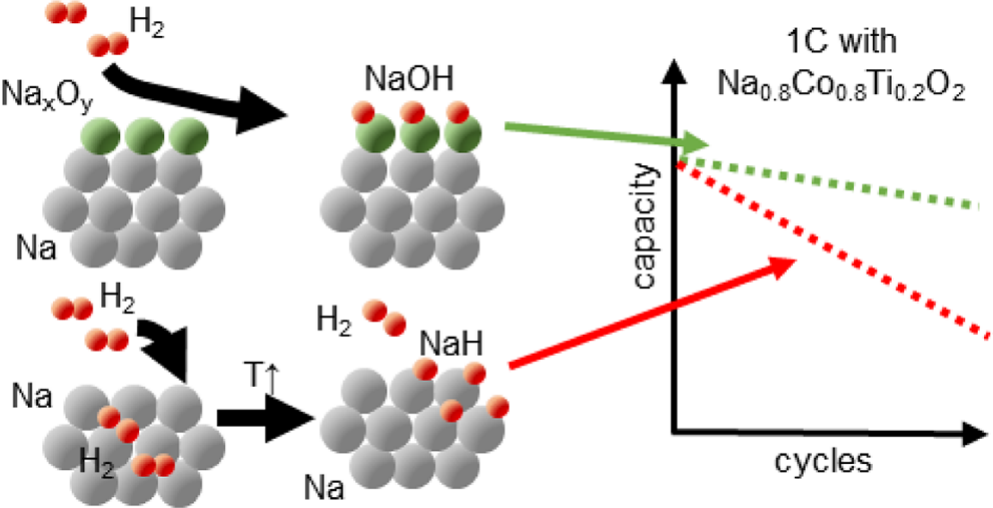

Secondary reactions and solid-electrolyte interface (SEI) formation are crucial aspects for battery lifetime. We show that one part of a natural SEI consists of crystalline NaH, which is formed on the sodium surface when carbonate-based electrolytes are used. Its impact on the electrochemical performance was studied using room-temperature H_2_-treated Na anodes and a NaH-Na composite anode. Depending on the preparation conditions, hydrogen was stored on the Na surface in the form of NaOH, enhancing the long-term performance of the cell with a layered Na-oxide cathode, or in the form of NaH, deteriorating the performance in comparison to a reference Na cell. With the help of thermogravimetry coupled with mass spectrometry, we identified an explosion-like thermal decomposition of fatigued Na anodes above approximately 120 °C, but H_2_-treated anodes exhibited higher stability of 10–30 °C compared to the reference anode. The composite NaH-Na anode shows a lower electrochemical capacity but no thermally induced explosion. Therefore, for a highly reactive metallic sodium anode, an effective protective layer against liquid electrolyte components is necessary to achieve high capacities and stable long-term operation. This passivation layer must fulfill the requirement of inertness to hydrogen gas to ensure a long lifetime.

## 1 Introduction

Sodium-ion batteries (SIBs) are promising candidates for stationary energy storage due to raw material availability and low-cost battery production.^1,2^ To reach the maximum specific capacity that can be offered by an SIB, the use of metallic sodium is necessary.^3−5^ However, metallic sodium easily forms dendrites during battery cycling^6−9^ and reacts with electrolyte components, even prior to electrochemistry, by simple contact of a piece of sodium with electrolyte solutions.^10,11^

In an operating battery, chemical redox reactions such as electrolyte decomposition and solid-electrolyte interface (SEI) formation take place immediately. With SEI formation, a critical point of the cell chemistry is reached. As it is known, needle-like sodium particles grow and easily crack during cycling, thus providing fresh metallic surfaces. This leads not only to constant electrolyte decomposition during battery operation but also increases “dead” sodium and loss of contact with the current collector. Therefore, the passive amount of sodium increases, leading to further electrolyte decomposition.^12^

Studies of sodium behavior in ester-based electrolytes (organic carbonates) reveal that the formed SEI mostly consists of a mixture of inorganic and organic Na compounds like NaF, Na_2_CO_3_, Na_2_O, sodium acetate CH_3_COONa, and probably NaH, depending on the salt and electrolyte used.^13^ Among these products, NaF and NaH were proposed mostly to stabilize the cycling behavior of the battery, since the overpotential of the battery cell decreases, and further side reactions are minimized, as concluded from the Coulombic efficiency close to 100%.^14^ On the other hand, some publications^15^ claim that the presence of H_2_ in the cell and the formation of NaH are responsible for the degradation of sodium batteries with NaPF_6_-containing ester-based electrolytes (carbonates) and a metallic Na anode. H_2_ is identified as the key component for NaH formation and, therefore, the deterioration of electrochemical performance. This statement is based on comparisons to NaPF_6_-containing ether-based electrolytes, without observable NaH formation during cycling. As a potential source of H_2_, electrolyte decomposition during contact with metallic sodium, both before and during cycling, is discussed in the literature.^16^

Here, we need to consider the main differences in cell chemistries between NaPF_6_-containing ester- and ether-based electrolytes. In ester-based solutions, HF forms by hydrolysis of the NaPF_6_ salt due to the presence of water traces in solvents, even prior to electrochemical treatments.^17^ Further reaction pathways are probably very similar to the reactions between HF and Li in electrochemical Li cells.^18,19^ The developed HF reacts with metallic Na with the formation of NaF and H_2_. Further, H_2_ can react with a fresh Na surface, resulting in the formation of NaH. In ether-based electrolytes, fluorine is bonded to phosphorus more strongly, eventually becoming a component of the SEI in the form of PF_6_^–^.^20−22^

So far, there are two different hypotheses addressing the effect of NaH formation in sodium batteries, without a detailed explanation of how NaH forms in the battery cell.

The formation of NaH from Na and H_2_ is a well-studied process known for more than a hundred years.^23−29^ The reaction of Na and H_2_ to form NaH requires temperatures above 200 °C^24^ due to kinetic reasons, since from the thermodynamic point of view, the reaction is exothermic with a negative reaction entropy value. Increasing H_2_ pressure above 1 bar facilitates the reaction.^30^ According to the Na–H phase diagram, NaH is the sole phase existing in the Na–H system.^31^

Solid sodium can also store hydrogen without NaH formation; its H_2_ storage capability was first reported in the year 1811^32^ and again in 1902.^33^ Further studies of hydrogen storage with sodium participation were conducted based on the Na–O–H^34^ and Mg–Na systems.^35^ A description of hydrogen storage within an electrochemical cell was provided for a K–Na alloy.^36^ The storage mechanism implies either hydrogen absorption or a reversible chemical reaction between sodium-containing components, such as a reaction of Na_2_O with H_2_ to form NaOH.^37^

Therefore, the property of sodium to store and release hydrogen provides new insight into possible hydrogen evolution during the charging and discharging of a battery. Additionally, not only NaH but also NaOH and Na_2_O as possible reacting partners with hydrogen, play an important role in the surface processes of metallic sodium during electrochemistry.

In the present work, we focus on the influence of stored hydrogen in a sodium metal anode on the electrochemical performance of sodium battery cells using a versatile approach.

First, we study phase formation on the Na surface in dedicated symmetric electrochemical cells with an excessive amount of electrolyte.

Second, we investigate the electrochemical performance of H-containing materials as anodes in Na batteries. The materials are prepared by room-temperature exposure of metallic Na chips to 10 bar of hydrogen in a pressure chamber.

Additionally, a commercial NaH powdered sample is tested in Na cells. Instead of NaPF_6_, we used NaClO_4_ as an electrolyte salt, dissolved in organic carbonates, since very little information in the literature is available for NaClO_4_-containing electrolyte formulations.

Third, we used thermogravimetry/differential thermal analysis coupled with mass spectrometry as a very sensitive and reliable method to detect hydrogen in the material. The obtained results point to a spontaneous formation of noticeable amounts of NaH on the Na surface during the operation of symmetric Na–Na cells.

Being used as an anode, NaH shows considerable electrochemical inertness. Furthermore, the artificial incorporation of hydrogen into the subsurface region of metallic sodium leads, among other things, to the formation of a NaOH layer, probably due to the presence of some unavoidable Na oxides on the pristine Na surface. A positive impact of the NaOH surface layer on the electrochemical performance of Na, known from previous work,^11^ is less pronounced here due to the extremely insulating nature of NaH, which is present in a major amount on the Na surface together with NaOH after exposure under 10 bar H_2_.^38^

## 2 Materials and Methods

### 2.1 Sodium

Sodium was used from a purchased sodium block or as a sodium chip. Sodium from an oil-passivated sodium block (Alfa Aesar, 99.95%) was cut into pieces and washed with heptane (Acros Organics, extra dry <0.005% H_2_O). After drying, a fresh sodium surface was obtained by removing the existing light gray layer until a metallic sheen appeared. For investigations, a small piece of sodium was rolled on a nickel current collector.

Already processed sodium metal was used as a chip (aot battery, 99.7%) coated on aluminum foil. The diameter of these chips is nearly 16 mm, with a chip thickness between 300 and 500 μm.

### 2.2 Sodium Exposure in Hydrogen

Sodium chips were stored for 1 week in a hermetically sealed container with a controlled hydrogen atmosphere at room temperature. During this period, a pressure of 10 bar of H_2_ was applied to the sodium chips. The H_2_-treated chips were transferred into an argon-filled glovebox (O_2_ < 0.1 ppm, H_2_O < 0.1 ppm) for further studies.

### 2.3 Structural Characterization

X-ray diffraction measurements were performed with a laboratory STOE STADI/P powder diffractometer, using monochromatic Mo–Kα_1_ radiation (λ = 0.7093 Å) or Cu–Kα_1_ radiation (λ = 1.54056 Å).

For measurements, all samples containing sodium were sealed with Kapton, which allows a measurement time of nearly 3 h, before characteristic NaOH reflections appear. The formation of NaOH on top of sodium is a clear signal of partial contact with the atmosphere. The sample of sodium after hydrogen exposure was sealed between low-density polyethylene (LDPE) to prevent direct contact with the Kapton foil and a subsequent reaction of the surface with the glue of the Kapton foil.

Tert-butanol (AppliChem, >99.5%) was used to separate NaH and Na from species that do not react with tert-butanol and are not soluble in it, such as NaCl or other residues that may form during cycling. For this, the electrodes were placed into tert-butanol after cycling. The remaining powder after filtration was then analyzed by XRD.

Microscopy measurements were conducted using a digital Keyence VHX-6000 microscope in the argon-filled glovebox (O_2_ < 0.1 ppm, H_2_O < 0.1 ppm).

### 2.4 Electrochemical Investigations

Electrochemical investigations were performed in Swagelok-type cells. As a separator, two layers of Whatman glass fiber were used. For experiments without a separator, two seal rings were used instead of the separator, thus creating a free volume of around 125 μL between the two electrodes. During the assembly of the Swagelok cells, less pressure than usual was applied to avoid a short circuit of the cell. The electrolyte used was 1 M NaClO_4_ in ethylene carbonate/propylene carbonate (EC/PC) in a ratio of 1:1. NaH (Sigma-Aldrich, 95%) was tested as an anode by mixing NaH with THF (Sigma-Aldrich, ≥99.9%, anhydrous) or as a slurry with conductive C65 carbon (Timrex C-Term, > 95%) and PTFE (Sigma-Aldrich) as a binder. These mixtures were dropped onto the nickel current collector. After THF evaporation, a thin film of NaH or a NaH/C/PTFE mixture appeared.

The battery cycling was performed galvanostatically in a glovebox with a constant current rate using a VMP3 Potentiostat (Bio-Logic SAS, Claix, France) at room temperature.

Symmetric Na∥Na cells and half-cell NaH∥Na or Na (H_2_ loaded)∥Na were cycled with a current density of 1 mA/cm^2^ for a capacity of 1 mAh/cm^2^. Full cells, with a Na_0.8_Co_0.8_Ti_0.2_O_2_ cathode^39,40^ and various Na-containing anodes (Na as a reference, H_2_-loaded Na, NaH-Na composite, or NaH), were cycled with a 1 C current rate (256 mA/g), determined by the mass of the cathode active material.

The Na_0.8_Co_0.8_Ti_0.2_O_2_ active material was mixed with the conductive additive C65 and the PVDF binder in a mass ratio of 80%:10%:10%. The mixture in *N*-methyl-pyrrolidone was cast onto aluminum foil using a doctor blade technique. The cycling took place in Swagelok cells at room temperature in an argon-filled glovebox or in a thermally isolated cupboard.

### 2.5 Thermogravimetry Coupled with Mass Spectrometry (TG-MS)

Thermal behavior of various pristine and cycled samples (sodium from the metal piece, initial Na chip, Na chip after loading with hydrogen, and Na chip after electrochemical cycling in a battery cell) was determined by differential thermogravimetric analysis (TG-DTA) and subsequent qualitative and quantitative analysis of the gas phase. Purchased NaH was used as a standard for quantifying the hydrogen.

The composition of the gas phase was analyzed as a temperature-dependent process using a TG-MS system, which is a combination of a thermo-balance STA 409 CD (NETZSCH) and a quadrupole mass spectrometer QMS 422 (Pfeiffer Vacuum). The installation of this system in an argon-filled glovebox (MBraun) enables handling and measurement of especially air- and/or moisture-sensitive samples.

The individual samples were measured under the following conditions: a temperature range of 25 to 450 °C (or up to 600 °C) for NaH of 25 to 500 °C; a sample mass of approximately 40–50 mg, for NaH of 1–5 mg, and for fatigued Na anodes of 10–12 mg; and a rate of heating and cooling of 5 K/min. A corundum crucible in a Knudsen cell with a Ta inlay and a perforated lid (effusion bore diameter 0.2 mm) with a type S (PtRh/Pt) thermocouple was used. The measurements were carried out in a flowing argon atmosphere as purging gas (Ar 99.999%, 50 mL/min with subsequent drying and oxygen postpurification via Big Oxygen Trap by Trigon Technologies). The detection of gas species was carried out in MID mode for ions with *m*/*z* 1 (H^+^), 2 (H_2_^+^), 16 (O^+^), 17 (HO^+^), 18 (H_2_O^+^), 32 (O_2_^+^) and additionally for the sample cycled in the battery, 28 (CO^+^), 35 (Cl^+^), 44 (CO_2_^+^), and 70 (Cl_2_^+^) with 70 eV electron impact ionization.

During the entire heat treatment, the gases evolving from the specimen were intermixed with a continuous argon purging gas stream and directed to the ionization chamber of the mass spectrometer by using a skimmer located directly above the bore of the effusion crucible. Background mass spectra were recorded before the measurement by using the same conditions. A buoyancy correction of the TG signal was not carried out in each case, as the exact mass change in dependence on the temperature was not the direct focus of interest, and in this case, a different crucible selection would have been more advantageous.

The quantification of the released hydrogen was carried out by calibration using four independent measurements on the NaH reference (Sigma-Aldrich, 95%). The chemical composition was determined by carrier gas hot extraction with 3.7 wt % and used as a base value.^41^

## 3 Results

### 3.1 Phase Formation on the Na Electrode during Repeated Na Plating and Stripping

**Figure 1:**
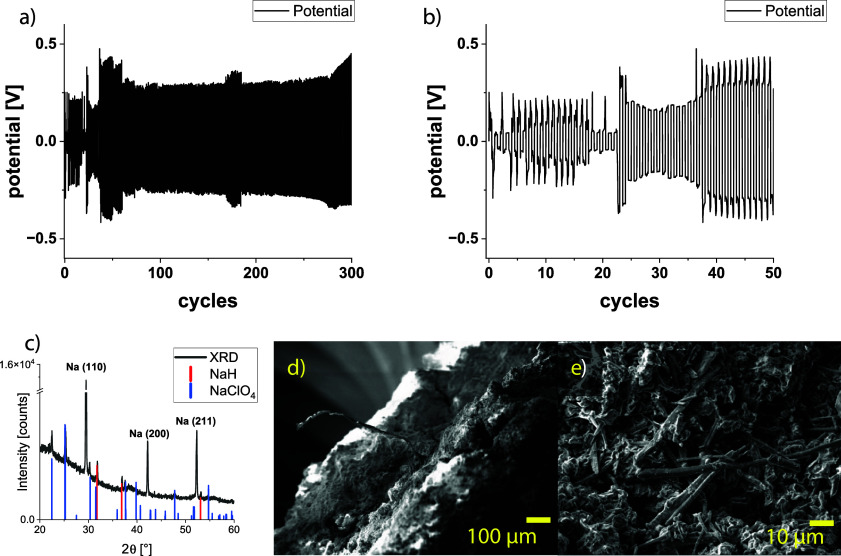
(a–b) GCPL of a symmetric separator-free sodium Swagelok cell, cycled at 1 mA/cm^2^ for 1 h. (c) XRD pattern (Cu–Kα_1_ radiation) of a Na layer removed from the surface of the Na electrode. (d–e) SEM images of the Na electrode after long-term cycling in a separator-free symmetric Swagelok cell.

In order to identify crystalline phases formed on the metallic Na electrode during battery operation, a symmetric Na∥Na electrochemical cell was cycled for a long time, followed by the XRD analysis of the electrodes. The isolation of the Na chip from the glass fiber separator is always challenging due to the intergrowth of Na-dendrites in the separator. To overcome this issue, a special separator-free Na∥Na Swagelok cell with two seal rings for the isolation of the electrodes was used. The cell was cycled for 300 cycles (about 600 h). Starting from the 50th cycle, a nearly stable sodium deposition and stripping can be concluded, based on the stable cell potential evolution (Figure 1a,b).

After cycling, a well-developed surface layer containing fibrous platelets and needles formed on top of the metallic sodium (see Figure 1d,e).

According to the XRD measurement (Figure 1c), the sodium electrode after long-term cycling consists of Na (*Im*3̅*m, a* = 4.28 Å),^42^ NaH (*Fm*-3*m*, *a* = 4.89 Å),^43^ and NaClO_4_ (*Cmcm, a* = 6.80 Å, *b* = 6.34 Å, *c* = 6.64 Å),^44^ most likely as residual electrolyte after evaporation; for detailed information, see Section 1 of the Supporting Information and Figure S1. The formation of other cubic phases such as NaCl (*Fm*-3*m*, *a* = 5.63 Å),^45^ NaO_2_ (*Fm*-3*m*, *a* = 5.51 Å),^46^ or Na_2_O (*Fm*-3*m*, *a* = 5.56 Å),^47^ which might be present on the Na surface as well, can be unambiguously excluded. The decision about the presence of NaCl is challenging. Due to the overlap of the 200 reflection of NaCl and the 111 reflection of NaH, it is necessary to secure whether the 200 reflection of NaH or the 220 reflection of NaCl is present, since only with this information can both phases be distinguished (see Figure S1a). Another possibility to differentiate between NaH and NaCl is the separation of the mixture with tert-butanol. Tert-butanol dissolves NaH but not NaCl. In our case, no NaCl was present after solvation. Therefore, the measured reflections can clearly be ascribed to NaH, according to these two identification methods (see Figure S1b,c).

The compounds NaH and NaClO_4_ are electronic insulators and most likely act as a solid protective layer on the Na surface because the formed surface layers on both electrodes were thick enough to bring in contact both electrodes, but no short circuit was detected. Using this separator-free symmetric cell enabled proving the formation of crystalline NaH on the sodium surface during cycling. A simple storage of metallic Na in the 1 M NaClO_4_ electrolyte solution did not lead to NaH formation, as shown in the previous work^11^ (for additional information, see Section 2 of the Supporting Information).

### 3.2 Sodium before and after Hydrogen Treatment

Formation of NaH on the metallic Na anode during repeated Na deposition and stripping encouraged us to investigate the role of NaH in Na cells during battery operation. For this, we decided to create an artificial NaH layer on sodium chips through their storage under 10 bar H_2_ at room temperature for 1 week. From the thermodynamic point of view, the Gibbs energy Δ_r_G°_298_ for the reaction Na_(s)_ + 1/2H_2(g)_ → NaH_(s)_ is strongly negative (−33.6 kJ mol^–1^),^48^ corresponding to an equilibrium H_2_ pressure over the phase mixture of solid metallic Na and solid NaH of approximately 10^–12^ bar at 298 K.^49^ Therefore, only a low reaction rate of hydrogen intercalation would prevent a complete transformation of Na into NaH.

**Figure 2:**
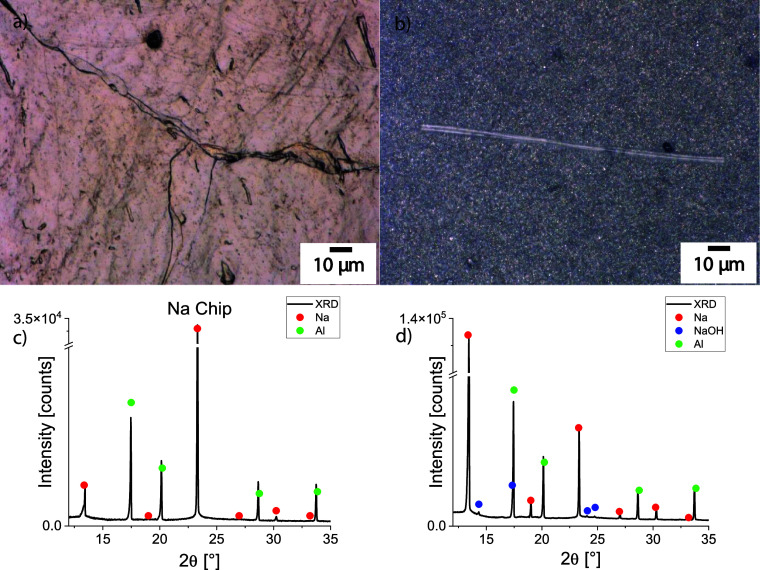
(a–b) Digital microscopy images of pristine metallic sodium chip (a) and after exposure to H_2_ for 1 week (b). The brownish, rough surface (a) becomes homogeneous and gray with a lot of small particles (b). A transparent fiber is clearly visible, which could be NaH formed during H_2_ treatment. (c–d) XRD of a Na chip before (c) and after exposure to H_2_ for 1 week (d), Mo–Kα_1_ radiation. Reflections of Na (red points) and Al (green points) from the Al layer on the bottom of sodium chips are present in both samples. After H_2_ storage, the Na chip shows reflections of NaOH (blue points), but not of NaH.

Digital microscopy of a fresh sodium chip and a sodium chip exposed to a H_2_ atmosphere clearly shows the influence of hydrogen on the sodium surface (Figure 2).

The virgin surface of sodium appears brownish under the light microscope and is rough, with pits, pores, and craters. In contrast, the H_2_-treated sodium surface is smoother, containing, however, a lot of small particles, shining in different colors. The presence of transparent fibers is an indicator that metallic sodium fibers react with hydrogen, forming NaH, which could be transparent.^50^ This surface is stable in an argon-filled glovebox but is surprisingly not stable in contact with Kapton, which is usually used to seal air-sensitive samples for XRD. A fast color change, observed by sealing the sample between pieces of Kapton foil, forced us to use the LDPE foil as additional protection. Since NaH does not react with Kapton (Figure S3), we assume that some amount of amorphous NaH, or a solid solution representing hydrogen dissolved in metallic Na,^49^ or even nanocrystalline sodium could be present on the Na surface after H_2_ treatment, which can be the reason for sample instability after contact with Kapton.

According to the XRD analysis, the surface of virgin sodium is free from any crystalline species beyond Na and Al, present as a thin layer on the backside of Na (Figure 2c). The main phase of the treated sample represents metallic sodium as well. However, there is another distribution in intensities of Bragg reflections, pointing to some recrystallization of Na crystallites. Moreover, the XRD pattern of treated sodium shows some amount of crystalline NaOH. No traces of NaH, Na_2_O or Na_2_O_2_ were detected (Figure 2d). Probably, metallic sodium, even if it was stored in an argon-filled glovebox, has some oxygen on the surface.^11,51^ In this case, amorphous sodium oxides can react with hydrogen to form NaOH. For example, calculations of the Gibbs energy Δ_r_G°_298_ for the reaction 1/2Na_2_O_2(s)_ + 1/2H_2(g)_ → NaOH_(s)_ result in the value of −155 kJ mol^–1^, a value significantly smaller than Δ_r_G°_298_ = −33.6 kJ mol^–1^ for the reaction Na_(s)_ + 1/2H_2(g)_ → NaH_(s)_.^48^

The other possible secondary reaction suggests the presence of some gaseous water traces in the commercial H_2_ gas container as a reason for the NaOH formation: Na_(s)_ + H_2_O_(g)_ → NaOH_(s)_ + 1/2H_2(g)_. The calculated Δ_r_G°_298_ value of −151.2 kJ mol^–1^ for this reaction is negative as well.^48^ Although some transparent fibers on the Na surface are quite long (>10 μm) and, therefore, must be detectable by XRD, their overall amount could be too low to give signals in the XRD pattern. Interestingly, the second H_2_ exposure experiment did not show any NaOH formation on the Na surface (see Section 4 of the Supporting Information and Figure S4 for more details). Therefore, the formation of NaOH strongly depends on the species present on the surface of bare sodium chips and the purity of the H_2_ source.

### 3.3 Analysis of Hydrogen Storage in Metallic Sodium and NaH

**Figure 3:**
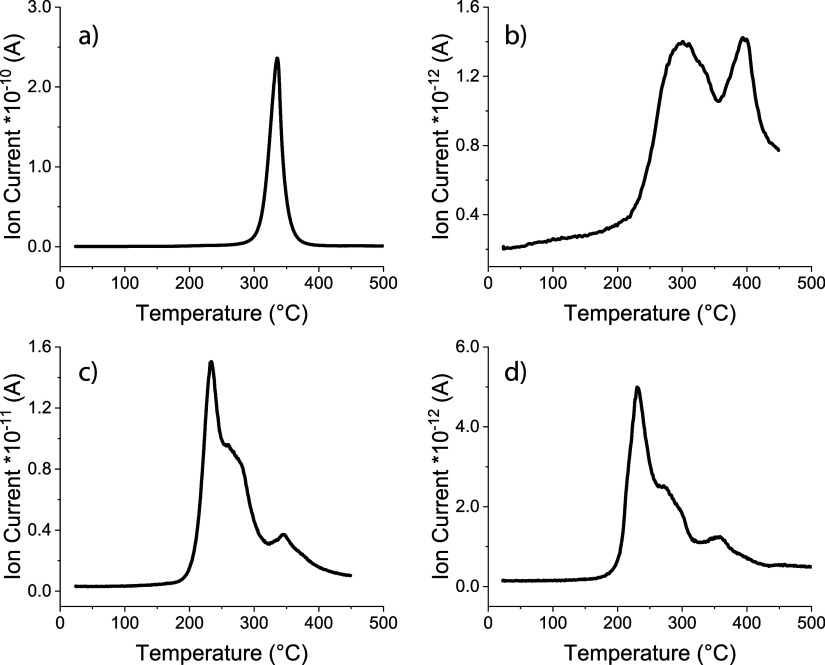
TG-MS analysis (detection of H_2_^+^ ions) of: (a) commercial NaH, (b) pristine sodium chip, (c) sodium chip after exposure to H_2_ during 1 week, with a NaOH-dominated surface, and (d) sodium chip after exposure to air during several seconds.^11^ It is apparent that pristine and H_2_-treated sodium metal implies various hydrogen-containing species including NaH, which decomposes in the temperature range between 300 and 350 °C.

Incorporation of hydrogen into metallic sodium was systematically studied using thermogravimetry coupled with mass spectrometry (MS) (Figure 3). The amount of hydrogen in the sample was calculated based on a calibration curve, constructed as a direct dependence of ion current on the mass of the NaH reference material (Figure S5). The thermal decomposition of NaH shows only one sharp, well-reproducible, and very well-quantifiable signal in the MS spectrum for the release of hydrogen into the gas phase between 300 and 380 °C with the maximum at 335 °C (Figure 3a). The decomposition behavior, peak shape, baseline, and reproducibility are suitable for using the hydrogen released during the thermal NaH decomposition, for quantitative calibration, despite the lower amount of H experimentally determined in NaH: 3.7(1) wt % vs 4.17 wt % from the theory, based on the NaH composition. We determined the hydrogen content in purchased sodium chips in the pristine state, after exposure under H_2_ atmosphere, after storage of Na in air for several seconds leading to the formation of NaOH on the surface,^11^ and in commercial NaH as a reference. The TG-MS analysis of sodium chips used for the experiments shows small amounts (∼0.003 wt % H_2_) of hydrogen (*m*/*z* = 2), see also Table 1.

**Table 1:** Results of TG-MS Measurements of Various Pristine and Cycled Sodium Materials (150–160 Cycles) with Temperatures of Certain Species Evolution: Hydrogen (*m*/*z* = 2), Oxygen (*m*/*z* = 16, 32), Water (*m*/*z* = 17, 18), Carbon Monoxide, and Carbon Dioxide (*m*/*z* = 28, 44)^a^

Material	*T* of H_2_ evolution (°C)	H_2_ content (wt %)	*T* of O_2_ evolution (°C)	*T* of CO/CO_2_ evolution (°C)
NaH reference	340	3.7(1)	–	–
Na chip reference	290(max), 390	0.0034	290, 380	–
Na chip after air storage for 5 s.	235(max), 280, 350	0.043	200–240	–
Na chip after H_2_ loading with NaOH formation	230(max), 270, 350	0.026	260	–
Na chip cycled, H_2_ loading with NaOH formation (20 cycles)	130^b^	1.008	130^b^	100, 130^b^
Na chip cycled, H_2_ loading with NaH formation	155^b^	1.56	155^b^	95, 155^b^
Cycled composite NaH(powder)-Na anode	150, 250(max), 290, 350	0.545	150, 260, 290, 350	90–115(max), 150, 210
Na chip cycled as reference	125^b^	0.947	125^b^	90, 125^b^

a Chlorine (*m*/*z* = 35, 70) was detected in very small amounts in cycled samples.

b Explosion-like thermal decomposition.

It is released to the gas phase in two steps, starting at 210 °C with a maximum at 295 °C and a further maximum at 390 °C (Figure 3b). Both temperatures lie above the melting point of sodium at approximately 98 °C;^52^ see also Figure S6a. The release of traces of oxygen (*m*/*z* = 16) can also be detected in this range (Figure S6b). This type of hydrogen release as a function of temperature can be interpreted as the existence of differently chemically bound hydrogen in sodium. At the same time, some chemical reactions involving molten Na cannot be omitted. For example, a reaction Na_(l)_ + NaOH_(s)_ → Na_2_O_(s)_ + 1/2H_2(g)_ cannot be excluded, because it takes place between 200 and 300 °C.^37^ A negative value of −4.8 kJ mol^–1^ for the calculated Gibbs energy Δ_r_*G*°_T_ of this reaction at 300 °C confirms its thermodynamic possibility.

Above 430 °C, the clearly measurable vaporization of sodium begins (Figure S6b).

The sample loaded with hydrogen contains approximately 0.026 wt % H_2_, significantly more hydrogen than the starting material, as expected (Figure 3c). This clearly shows that sodium can uptake an additional amount of hydrogen over a certain period of time. The release essentially takes place in the same temperature range in three stages. In the temperature range of 180–320 °C, as mentioned above, the hydrogen release could also be a result of the reaction between liquid Na and NaOH with the formation of Na_2_O and H_2_ or a result of the reaction between NaH and NaOH with the formation of Na_2_O and H_2_: NaH_(s)_ + NaOH_(s)_ → Na_2_O_(s)_ + H_2(g)_.^37^ Although the calculated Gibbs energy Δ_r_*G*°_T_ for this reaction at 300 °C is slightly positive (+6.4 kJ mol^–1^), vacuum conditions would shift the reaction toward products. Since NaOH is present at the surface after exposure to hydrogen, these reactions must be taken into consideration. A well-separated peak with the maximum at 345 °C can be ascribed to the thermal decomposition of NaH. Additionally, the thermal behavior of NaOH under the same conditions was studied. For this, two NaOH materials, a commercially available water-containing one and a dried NaOH material were used (Figure S6c,d). Only water evaporation up to 250 °C was observed for the water-containing NaOH, in good agreement with the literature,^53^ while the dried NaOH sample is stable up to 850 °C.

Interestingly, the Na sample after 5 s exposure in air shows a very similar three-stage H_2_ release behavior to the sample after H_2_ loading with a NaOH-dominated surface (Figure 3d). It contains even more hydrogen (0.043 wt %), and the third stage around 350 °C corresponds to the decomposition of NaH, probably present in Na initially. Additionally, the amount of NaH in the sample could accumulate at temperatures below 300 °C due to the solid-state reaction NaOH_(s)_ + 2Na_(l)_ → NaH_(s)_ + Na_2_O_(s)_, which is possible from the thermodynamic point of view: Δ_r_*G*°_T_ = −9.61 kJ mol^–1^ at 250 °C.

The oxygen value is nearly the same for all three samples within measurement accuracy.

Therefore, in a sodium battery, metallic sodium represents a possible hydrogen source (although in low quantities), and hydrogen can be stored in or on the surface of metallic sodium. The surface storage in this case should be considered in combination with other reaction products of metallic sodium, especially those with NaOH.

### 3.4 Electrochemical Behavior of NaH and H_2_-loaded Sodium as Anodes

Commercial NaH, a NaH-Na composite, and H_2_-loaded Na chips were tested as negative electrodes in Na cells with a Na_0.8_Co_0.8_Ti_0.2_O_2_ cathode. The redox potential of the H_2_/2H^–^ pair of −2.2 V vs the standard hydrogen electrode (SHE)^54^ is higher than that of the Na^+^/Na redox pair (−2.71 V). Therefore, NaH could theoretically serve as an anode in Na batteries if a satisfactory solubility of gaseous H_2_ in the electrolyte solution is ensured.

We did not find sufficient literature about the dissolution of H_2_ in liquid organic carbonates, besides a contribution where a value of 0.055 μg H_2_ dissolved in 1 mL EC/DMC/DEC solvent at 25 °C after 30 min was reported.^55^ This value is about 1000 times lower than what would be required for the redox reaction with Na-oxide cathodes in conventional Swagelok cells.

From this, we can conclude that a reversible redox activity of NaH is rather not to be expected. Another aspect negatively influencing the redox activity is its very low electronic conductivity: the calculated band gap of NaH is 3.77 eV.^56^

According to the Na–H phase diagram, NaH represents a single binary phase with a very narrow homogeneity range.^57^ Therefore, the cell with NaH as an anode should be considered as a “zero-access” Na battery, and the impact of bulk NaH on the stripping/platting behavior of Na can be evaluated. In the battery with the H_2_-loaded Na, NaH and NaOH on the Na surface represent an artificial layer, influencing the deposition behavior of Na as well.

To evaluate the electrochemical properties of pure NaH, we studied the electrochemical behavior of a commercial NaH material as the next step.

#### 3.4.1 NaH Powder

**Figure 4:**
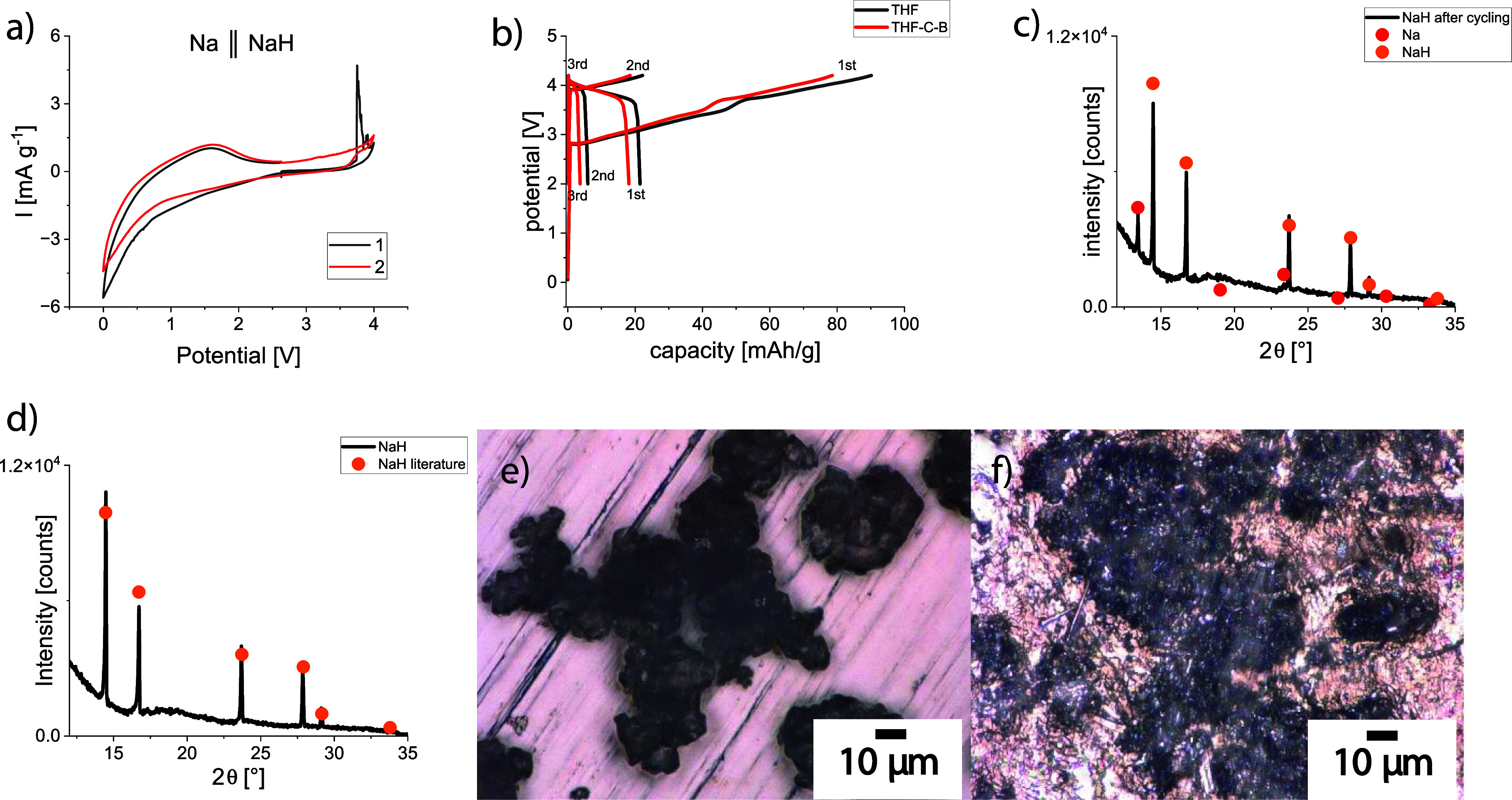
(a) Cyclic voltammetry (CV) of a Na∥NaH half-cell showing electrochemical NaH decomposition in the first cycle above 3.7 V. (b) Charging and discharging of a cell with a NaH anode and Na_0.8_Co_0.8_Ti_0.2_O_2_ cathode. Black line: NaH was coated onto the current collector from a THF solution. Red line: the electrode represents a mixture of NaH, conductive carbon, and binder. After cycling, metallic sodium is present in the NaH anode (c), while there is no sodium present in pristine NaH (d). THF-coated NaH on the Al current collector before cycling (e). NaH after cycling in the battery (f).

First, a cyclic voltammetry (CV) measurement of the half-cell Na∥NaH was performed. The OCV value of approximately 2.5 V vs Na^+^/Na is much higher than expected from the estimated literature value for the H_2_/2H^–^ redox pair,^54^ reflecting a high internal resistance in the battery. In the CV measurement, an irreversible oxidative peak in the first cycle above approximately 3.7 V vs Na^+^/Na is clearly seen, which probably corresponds to the decomposition of NaH (Figure 4a). A significant deviation from the thermodynamically proposed values points to a kinetic inertness of commercial NaH material.

Next, a battery with a NaH anode and a Na_0.8_Co_0.8_Ti_0.2_O_2_ cathode shows a cell potential close to zero under OCV conditions. The cell potential increases immediately after applying a positive current during charge, achieving a value of 2.5 V, usually observed in batteries with a Na_0.8_Co_0.8_Ti_0.2_O_2_ cathode and metallic Na anode.^40^ This confirms the redox inertness of NaH: in the case of its possible activity, the cell potential should be about 0.5 V lower due to the lower redox potential of the H_2_/2H^–^ pair. During the first charge with a 256 mA/g current density (1C rate, calculated for the cathode), a capacity of 70–80 mAh/g can be obtained, which is comparable with the performance of cells with a metallic Na anode^40^ (Figure 4b). A first battery discharge results in a much lower capacity of about 20 mAh/g. Following cycles lead to capacities close to zero. Hence, sodium can be extracted from the cathode during the first charging, but the Na stripping from the anode side is hindered, leading to a totally incapable battery. XRD analysis of the anode side after several charge–discharge cycles revealed the presence of NaH and crystalline metallic Na (Figure 4c), although pristine NaH did not contain any crystalline Na (Figure 4d). Digital microscopy photographs of the anode before cycling showed an island-like distribution of aggregated NaH particles on the Al current collector (Figure 4e). After cycling, the particles are denser and more aggregated than before, and the deposited metallic Na is distributed on the anode surface, mostly between NaH particles (Figure 4f).

Obviously, a very low Coulombic efficiency of the NaH∥Na_0.8_Co_0.8_Ti_0.2_O_2_ cell arises from poor Na deposition reversibility, as known from the literature for carbonate-based electrolytes.^14^

**Figure 5:**
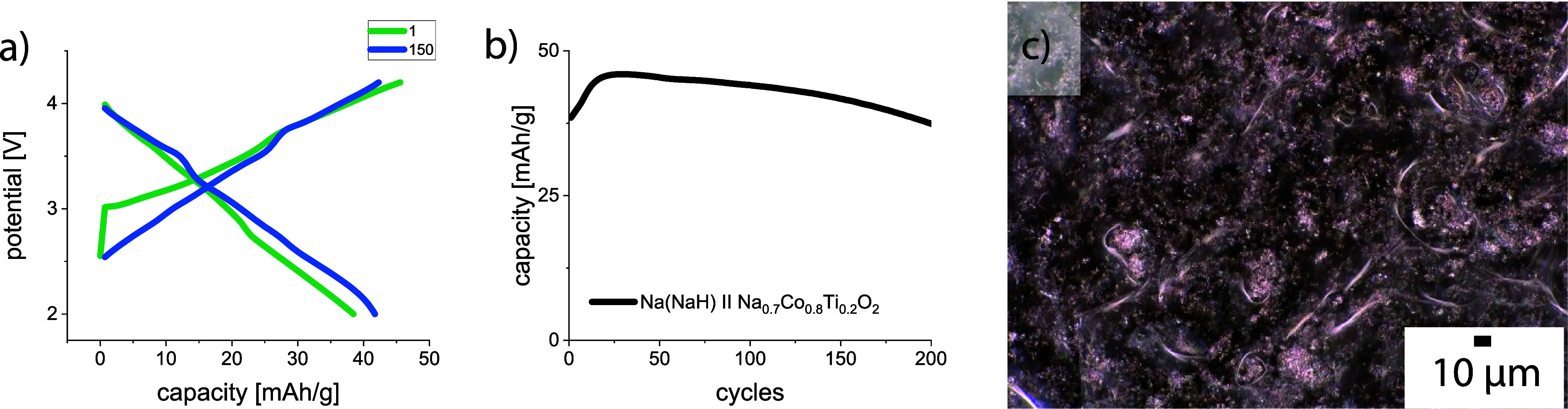
Potential profile (a) and discharge capacity vs cycle number (b) of a Na cell with the Na_0.8_Co_0.8_Ti_0.2_O_2_ cathode and a NaH-Na composite anode at 1 C current density (256 mA g^–1^). Digital microscopy after cycling (c) clearly shows a darkened surface with small particles but no signs for dendrite growth.

The creation of a composite anode containing a NaH powder layer on top of the metallic Na chip significantly improves its cycling stability in cells with the Na_0.8_Co_0.8_Ti_0.2_O_2_ cathode (Figure 5), although the absolute capacity values decrease drastically. In addition to its low electronic conductivity,^56^ NaH also suffers from low ionic conductivity, like all metal hydrides.^58,59^ On the one hand, the NaH powder used protects metallic sodium from direct contact with the electrolyte and minimizes dendrite growth, but on the other hand, it hinders ionic transport. Therefore, it also hinders Na plating, thus reducing the battery capacity performance. Dendrite growth becomes visible when the surface of the composite anode is investigated under a microscope after cycling. Besides a blackened surface with small particles, no typical dendrite structures are visible.

#### 3.4.2 Cycling Behavior of H_2_-loaded Sodium Anodes

In the case of modified Na anodes after exposure to a H_2_ atmosphere, battery cells with NaOH- and NaH-dominated Na surfaces should be considered separately. Previously,^11^ we showed a positive impact of the NaOH layer on top of metallic Na for the long-term electrochemical cycling of a Na-battery. Here, NaOH suppresses side reactions with the electrolyte and hinders Na recrystallization caused by the current flow.

**Figure 6:**
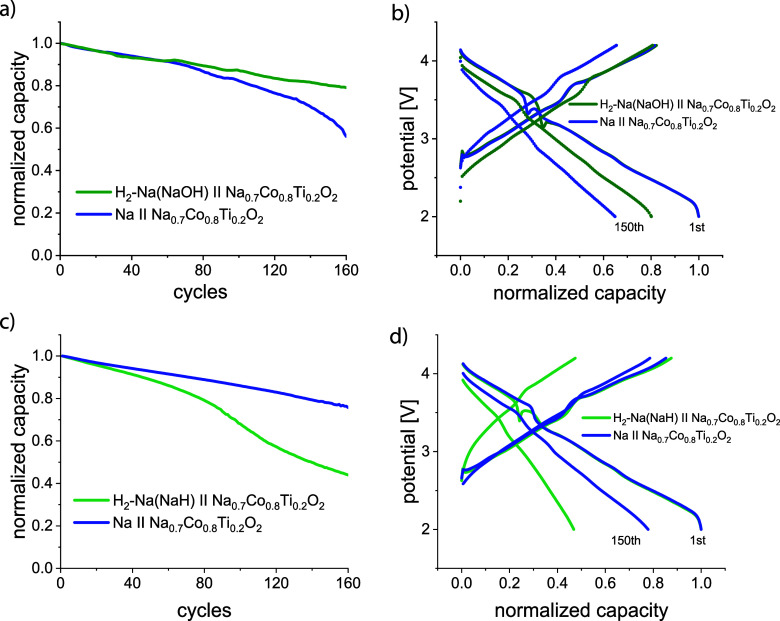
Discharge capacity vs cycle number and a potential profile of Na cells with Na_0.8_Co_0.8_Ti_0.2_O_2_ cathode and H_2_-loaded sodium anode with predominantly NaOH (a–b) and NaH (c–d) on the surface in comparison with a cell using untreated Na.

Creating a NaOH layer on the Na surface during sodium exposure under a H_2_ atmosphere for 1 week seems to have the same positive impact on the long-term stability of the Na battery. A cell built with the H_2_-loaded Na shows better cycling stability at a 1 C current density than a cell with untreated sodium, measured as a reference (Figure 6a).

The first charging and discharging are very similar for both batteries, except for a slightly higher discharge capacity of the cell with untreated Na. However, in the 150th cycle, the cell with untreated Na shows a bigger potential hysteresis of 0.67 V between charge and discharge, measured as a potential difference at a half capacity value, in comparison to 0.42 V in the cell with the H_2_-loaded Na with a NaOH surface. Apparently, the cell with untreated Na suffers from hindered Na plating and stripping, although the stripping features a higher resistance, also in the cell with the H_2_-loaded Na. The capacity in the 150th cycle is also noticeably higher for the H_2_-loaded Na cell.

If no NaOH is present on the surface of the H_2_-treated sodium, the cycling stability is significantly lower, even compared to a cell with untreated sodium (see Figure 6c,d). Interestingly, Na deposition and stripping in a half-cell Na (H_2_-loaded)∥Na do not show a significant difference in cycling stability compared to a symmetric Na∥Na cell (see section 7 of the Supporting Information for more details).

#### 3.4.3 Mass Spectrometric Analysis of Fatigued Sodium Anodes after Battery Operation

**Figure 7:**
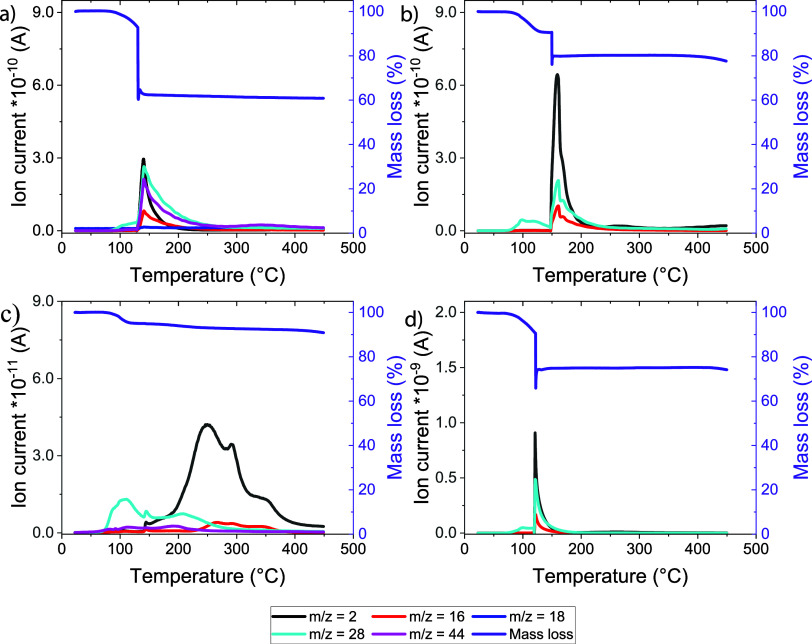
TG-MS measurements of various Na anodes after cycling in Na cells with the Na_0.8_Co_0.8_Ti_0.2_O_2_ cathode: (a) H_2_-exposed Na with a NaOH-dominated surface, (b) H_2_-exposed Na with a NaH-dominated surface, (c) NaH-Na composite anode, and (d) reference Na anode.

The impact of sodium modifications on the chemical composition of the solid electrolyte interface (SEI) after prolonged cycling with a Na_0.8_Co_0.8_Ti_0.2_O_2_ cathode was studied by TG-MS analysis. We used Na-chips from the batteries after operation, as shown in Figures 5 and 6. The Na samples after H_2_ exposure, as well as a reference Na chip, are characterized by an explosive decomposition (“thermal runaway”), recognizable by the oscillating weighing signal, with simultaneous evolution of hydrogen (*m*/*z* = 2), oxygen (*m*/*z* = 16, 32), water (*m*/*z* = 17, 18), carbon monoxide, and carbon dioxide (*m*/*z* = 28, 44), see Table 1 and Figure 7. The decomposition temperature is about 125–130 °C for the Na reference and NaOH-containing Na-chips and 20 °C higher for the H_2_-loaded Na chip without noticeable amounts of NaOH. In contrast, the NaH-Na composite sample demonstrated a much smoother thermal decomposition with gaseous species appearing successively in a broad temperature range (see Table 1). Note that the decomposition of all cycled samples starts with CO evolution (*m*/*z* = 28) already below 100 °C. The amount of volatile chlorine (*m*/*z* = 35, 70) in all samples is very tiny. Using the calibration curve, we could determine the exact amount of hydrogen in the materials: it increased by 40–200 times after cycling in the battery cell, achieving 1.56 wt % for the sodium chip after H_2_ loading without a noticeable amount of NaOH. Note that the NaH-Na composite anode with a smoother decomposition process exhibits much fewer H-containing species, resulting only in 0.545 wt % hydrogen.

Unfortunately, quantitative determination of carbon oxide and water amounts was not possible. However, the intensity of the ion current, which is proportional to the species quantity, decreases for cycled Na anodes in a similar way: hydrogen (*m*/*z* = 2), carbon monoxide (*m*/*z* = 28), and oxygen (*m*/*z* = 16). Only for the H_2_-loaded Na anode with NaOH, a large amount of carbon dioxide (*m*/*z* = 44) was detected, thus confirming more available oxygen in the sample.

The explosion-like decomposition could probably be initiated by the thermally induced redox reaction of NaClO_4_ from the surface with organic sodium carbonates, which represent a part of the solid-electrolyte interface (SEI), similarly to organic lithium carbonates.^60^ In this case, NaCl will remain in the sample, while oxygen reacts with organic fragments: NaClO_4(s)_ + C_*x*_H_*y*_O_z(s)_ → NaCl_(s)_ + CO/CO_2(g)_ + H_2_O_(g)_.

#### 3.4.4 *Operando* XRD Analysis of NaH-Containing Electrodes

Two different cells, one with the NaH anode and Na_0.8_Co_0.8_Ti_0.2_O_2_ cathode, and the other with NaH as a working electrode and metallic Na as a counter electrode, were studied using *operando* XRD analysis. In both cases, we focused on structural changes in the NaH-containing electrode.

**Figure 8:**
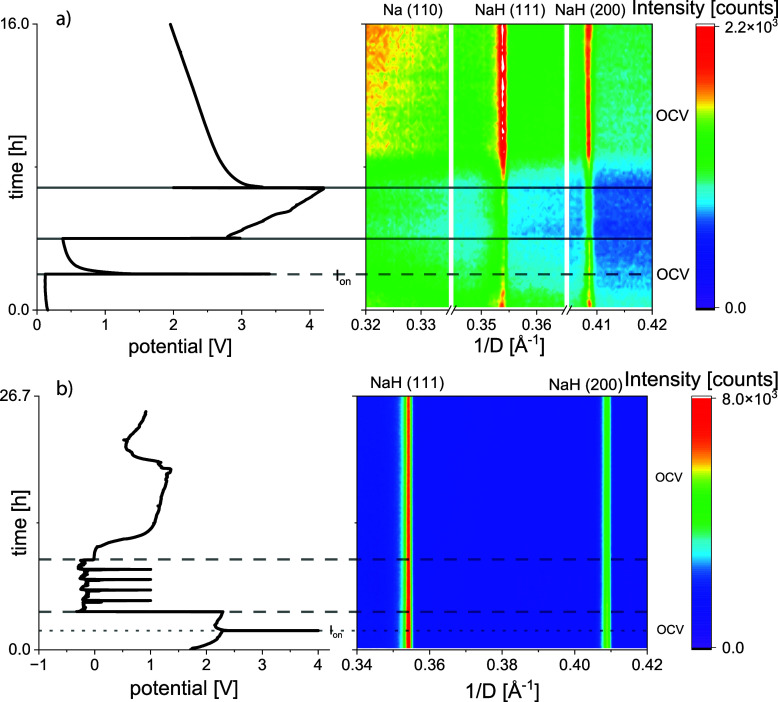
Electrochemical curves and corresponding XRD patterns as counter-plots of: (a) a NaH∥ Na_*x*_Co_0.8_Ti_0.2_O_2_ cell, and (b) a Na∥NaH cell with 1 M NaClO_4_ in EC/PC as electrolyte. In both cases, NaH is electrochemically almost inactive. Even if sodium is plated on the NaH side, there is no further stripping of the plated sodium.

The NaH∥Na_0.8_Co_0.8_Ti_0.2_O_2_ cell was first subjected to the open circuit voltage (OCV) regime for 2 h (Figure 8a), followed by a short positive current pulse of 1 mA/cm^2^ for 5 s with a subsequent relaxation time of 2 h, and galvanostatic charge/discharge with a constant current rate of C/10. The OCV regime during the 2 h before charge results in a stable cell potential of 0.2 V. However, the intensities of the (111) and (200) reflections of NaH were decreasing, pointing to some instability of the NaH structure in contact with the electrolyte solution (Figure 8a). A current pulse of 1 mA/cm^2^ for 5 s after OCV only slightly increased the cell voltage, without noticeable changes in the diffraction patterns. Therefore, no metallic Na is formed on the anode site since a potential of around 2.0–2.3 V would be expected for the cell with the Na_*x*_Co_0.8_Ti_0.2_O_2_ cathode and Na metal as a counter electrode. During galvanostatic charging, the electrochemical curve showed a development usually observed for cells with the Na_*x*_Co_0.8_Ti_0.2_O_2_ cathode and Na metal anode, although no crystalline sodium metal was detected. Probably, Na was deposited in an amorphous form or as nanoparticles. A further decrease in the intensities of NaH reflections was observed upon charging, but their position remained constant, pointing to zero changes in the cell metrics.

As expected, the cell could not be discharged at a relevant capacity. A subsequent charging with noteworthy capacities was therefore not possible. After charging/discharging five times, the cell was measured again under the OCV condition. A cell voltage of around 2 V was achieved for 8 h, with a tendency for further lowering. Interestingly, the intensities of NaH reflections and the background increased drastically, exceeding the initial values. At the moment, we do not have any satisfactory explanation for the observed phenomenon.

Apparently, some dissolution/decomposition and recrystallization of NaH could occur during galvanostatic processes in the cell. However, an additional experiment demonstrated a nearly zero NaH solubility in EC/PC, which was tested by storing NaH in EC/PC for 1 day at room temperature. Afterward, the solvent was filtered and eventually evaporated. Residuals were investigated with EDS, where no Na was detected.

Increasing reflection intensities of NaH could also be caused by the decomposition reaction of the electrolyte solvents with a noncrystalline and very reactive Na.

When NaH is cycled in a cell against metallic Na, the aqueous OCV regime gave values around 2 V (Figure 8b). A short positive current pulse of 1 mA/cm^2^ for 5 s raised the potential to 4 V and led to a small increase in the intensity of the (110) sodium peak, without changes to NaH.

Applying a negative current of 1 mA/cm^2^ led to sodium dissolution from the metallic sodium anode, and the potential dropped to nearly 0 V immediately. The following stripping of the plated sodium at the NaH side, after changing the current direction, did not take place. Some minor changes in the sodium peak intensity were observed. The intensities of the (111) and (200) reflections of NaH slightly decreased during the electrochemical treatment.

From the *operando* XRD measurements, we can conclude that the amount of NaH in the cell is repeatedly varying, but its crystal structure remains unchanged. When metallic Na is deposited on the negatively polarized electrode containing NaH, it cannot be stripped afterward. Therefore, insulating NaH probably significantly increases the internal resistance of the cell and promotes cell degeneration.

## 4 Discussion

We provided direct experimental evidence of NaH formation on metallic Na electrodes in electrochemical cells with a liquid electrolyte composed of a 1 M NaClO_4_ solution in EC/PC. The significant amount of NaH detected by XRD confirms redox processes with electrolyte components on the active Na surface during Na deposition and stripping upon battery operation, which, however, are suppressed by the sole contact of metallic Na with the electrolyte solution.

The evolution of gaseous hydrogen in the electrochemical cell, reported in the literature^16^ as a result of the presence of water and/or during electrochemical cycling, can easily lead to NaH formation on the sodium surface. Even if the amount of NaH is relatively low, it can negatively influence battery performance due to its strongly insulating nature and associated increase in cell resistivity, eventually leading to a rapid capacity decrease. Apparently, NaH forms on a fresh Na surface upon the Na deposition/dissolution process and accumulates over time. Additionally, other residual organic species/organic decomposition products accumulate over time and influence the long-term and thermal stability of the electrode.

In the literature, the long-term stability and performance of the metallic sodium anode in various electrolyte formulations were studied using mainly the NaPF_6_ salt.^15^ It was shown that the Na anode is more stable in ether-based electrolytes in comparison to ester-based (carbonates) ones.^15^ The unavoidable presence of water traces in carbonates causes hydrolysis of NaPF_6_, leading to the formation of HF and its reaction with metallic Na followed by H_2_ evolution.^17^ In the ether-based solvents, PF_6_^–^ anions are additionally stabilized due to the bonding to existing Na^+^(diglyme)_2_ complexes. Hence, the anion is stabilized, and hydrolysis to HF is mitigated.^20^ We showed that NaH formation occurs in carbonate-containing electrolytes with the NaClO_4_ salt, thus confirming the intrinsic instability of the carbonate solvents in a battery cell.

Target investigation of the NaH impact on electrochemical performance requires using artificial NaH in the Na anode. This was achieved in two ways: modification of the Na anode through its storage under H_2_ atmosphere, and creation of a composite Na anode with a NaH component. As a result of the high affinity of sodium for hydrogen, sodium exposed to hydrogen contains a higher amount of hydrogen than bare sodium. Depending on the surface composition of metallic Na, hydrogen can be stored as NaOH (if noticeable amounts of Na_2_O_2_ and Na_2_O are present on the surface), and in the form of NaH. Long-term stability tests of battery cells with a Na_0.8_Co_0.8_Ti_0.2_O_2_ cathode and metallic Na anodes after exposure to H_2_ convincingly demonstrated the advantage of a NaOH-dominated Na surface in comparison to a bare Na anode, in agreement with our previous findings.^11^ In contrast, a NaH-dominated Na surface of the Na anode acted rather negatively on the cycling stability, resulting in a much faster capacity loss than that of a reference cell.

A composite NaH/Na anode, prepared by pressing the NaH powder on top of metallic Na, showed lower capacity values calculated on the cathode basis (about 40% of the usual capacity value), which can be understood due to the reduced active Na surface and the insulating nature of NaH. The NaH powder itself does not participate in the main redox process. However, the capacity retention after more than 150 charge–discharge cycles was surprisingly high. Therefore, powdered NaH on top of metallic Na served as a protective barrier between electrolyte components and the Na metal, minimizing secondary redox reactions.

*Operando* XRD studies clearly showed variations in the amount of crystalline NaH in the cell.

A very important point is the thermal stability of the fatigued anode, which is directly related to battery safety. The surface composition of the Na anode seems to play a significant role here. An explosion-like thermal decomposition with severe mass loss (“thermal runaway”) was observed above 100 °C for the reference Na anode and Na anodes modified by exposure under H_2_ atmosphere, while a more controlled step-like decomposition with much lower mass loss was detected for the composite NaH/Na anode. Therefore, much fewer organic residuals, as a result of electrolyte decomposition, were deposited on the composite NaH/Na anode.

The reference Na anode shows the lowest thermal stability (temperature of explosion-like decomposition of 125 °C), followed by the Na anode with a NaOH-dominated surface (130 °C) and a NaH-dominated surface (155 °C). For all samples, the evolution of some amounts of CO was detected already above 95 °C. The composite NaH/Na anode does not show any thermally induced explosion.

The main decomposition products are hydrogen, carbon monoxide, and oxygen. In the case of the Na anode with the NaOH-dominated surface, a remarkable amount of CO_2_ (a higher oxidation state of carbon) was detected.

The obtained results disclose a very complex phenomenon at the solid-electrolyte interface between metallic Na and the electrolyte. Mechanical deposition of large (microsized) NaH particles on top of the Na anode prior to battery operation decreases cell capacity but avoids thermal explosion and enhances battery safety, although the surface chemical composition changes upon repeated electrochemical treatment. On the other hand, using a metallic Na anode with a NaH-dominated surface results in less stable cycling behavior of the cell. A thermally induced explosion of the anode was also observed.

## 5 Conclusion

The main conclusion of this work confirms the absolute necessity of protection for the sodium surface if metallic sodium is used as an anode in sodium batteries. The protective layer should hinder the reaction with organic carbonates during cycling. Using NaClO_4_ as the electrolyte salt also leads to the formation of NaH on the surface of the Na anode, similar to what is observed for carbonate-based electrolytes with NaPF_6_. The amount of NaH in the SEI seems to vary during cycling but mostly accumulates over time, together with other organic decomposition products. The low conductivity of NaH should contribute to higher internal resistivity in the battery cell. However, the thermal stability of the fatigued Na anode (and, therefore, the safety of the battery) is determined rather by residual organic species, not by the amount of NaH in the solid-electrolyte interface. Using NaH as artificial protection for the Na surface influences the cell chemistry positively regarding cycling and thermal stability, albeit at the cost of gravimetric capacity.

## Supporting Information Available

Analysis of NaH formation during cycling, additional information on sodium surfaces after H_2_ storage, calibration curve for H_2_ analysis with TG-MS, thermal behavior of sodium, additional electrochemistry (PDF)
