# Supplementary material for: Impact of NaH on the Electrochemical Performance of Sodium Batteries
Source: ACS Omega. 2025 Jan 14;10(3):2699–711. doi: 10.1021/acsomega.4c08310 (PMC11780441; doi:10.1021/acsomega.4c08310)
Supplement: Supplementary file 1 — ao4c08310_si_001.pdf [file ao4c08310_si_001.pdf]

Impact of NaH on the Electrochemical Performance of Sodium Batteries

Alexander Thomas,[*,†] Björn Pohle,[†] Marcus Schmidt,[‡] Henrik-Gerd Bischoff,[§] Marius Lau,[‖] Felix Heubner,[‖] Stefan Kaskel,[⊥] Daria Mikhailova [*,†]

[†] Leibniz Institute for Solid State and Materials Research (IFW) Dresden e. V., Helmholtzstraße 20, 01069 Dresden, Germany.

[‡] Chemische Metallkunde, Max-Planck-Institut für Chemische Physik fester Stoffe, Nöthnitzer Straße 40, 01187, Dresden, Germany.

[§] Schaufler-Professur für Kaelte-, Kryo- und Kompressorentechnik, Technische Universität Dresden, 01062 Dresden, Germany.

[‖] Fraunhofer Institute for Manufacturing Technology and Advanced Materials IFAM, Winterbergstraße 28, 01277 Dresden, Germany.

[⊥] Department of Inorganic Chemistry, Technische Universität Dresden, Bergstraße 66, 01069 Dresden, Germany.

Supporting Information

1. NaH formation during cycling in a Na II Na symmetric cell without separator

A repeated electrochemical sodium deposition and stripping in a Swagelok cell without separator and a following XRD analysis of Na-electrodes is a possibility to evaluate formation of NaH during cycling. A challenge for NaH detection is a similarity between its structure and the NaCl-structure (F m -3 m; a = 5.6338 Å) [1], which leads to an overlap of the first intense NaCl- and NaH-reflections in the XRD pattern (Figure S1a). Since the presence of NaCl cannot be excluded, a better separation between NaCl and NaH is necessary. For this tert-butanol can be used, since it reacts with NaH with Na_2O-formation, but not with NaCl. The solubility of NaCl in tert-butanol is also extremely low. For the analysis, the residuals after cycling were solved in tert-butanol, and non-soluble products were investigated with XRD.

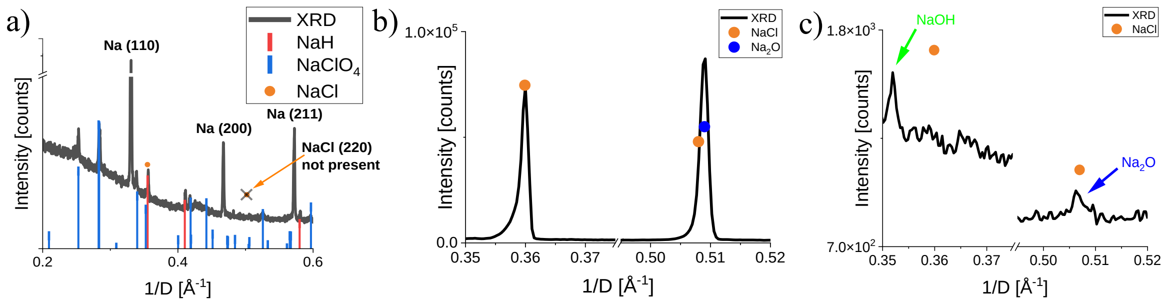

Figure S1. a) XRD of cycled sodium without separator. NaH (red) and $NaClO_4$ (orange) are clearly visible. The first reflection of NaCl (orange) overlaps with NaH, but the second NaCl peak is not detectable. b) NaCl and NaH solved in tert-butanol. NaH is solved and NaCl remains as a solid. Na_2O is also detectable. c) In comparison, when the cycled anode sample is solved in tert-butanol, the NaCl reflections are missing and only NaOH (green) and Na_2O (blue) are detectable.

For comparison, a mixture of NaCl and NaH was prepared and solved in tert-butanol (Figure S11b). As a result, only reflections of NaCl and Na_2O could be detected, thus confirming the possibility to separate NaCl and NaH with the solvent. Therefore, the cycled sample was solved in tert-butanol (Figure S1c). In this case no NaCl, but NaOH and Na_2O were detected. Since no NaCl is visible, we can conclude that NaCl is not present in the cycled sample, and the measured reflections clearly belong to NaH.

2. Sodium stored in 1 M NaClO4 in EC/PC for 5 d

To verify if NaH forms on top of metallic sodium when it is stored in 1 M $NaClO_4$ in EC/PC, the sodium piece was checked with XRD and digital microscopy after storage. Both methods revealed no signs for NaH formation (see Figure S2). Therefore, we can conclude that NaH does not form by simple storage of sodium in this electrolyte.

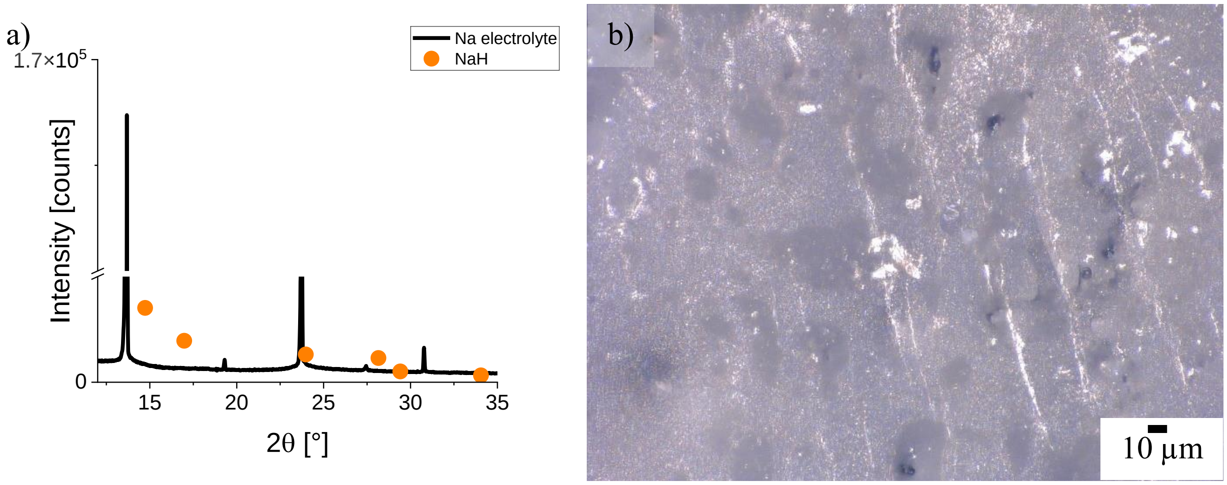

Figure S2. a) XRD and b) SEM of Na stored in 1 M $NaClO_4$ in EC/PC for 5 d, showing no NaH peaks or transparent structures on top of metallic sodium.

3. XRD's of NaH with and without Kapton contact

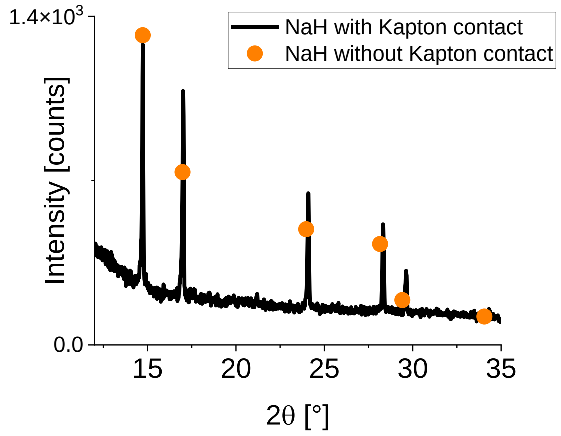

Figure S3. XRD patterns of a commercial NaH material with and without contact to Kapton foil. Only a difference in intensities of some reflections is observed, which occurs due to a preferred orientation of NaH particles, depending on sample preparation, like pressing the powder on Kapton.

4. Sodium surface after H_2-storage and storage under ambient conditions

A further comparison between a NaOH-dominated surface, formed on metallic sodium under ambient conditions during 5s, NaH-dominated surface and NaOH-dominated surface of the H_2-exposed sodium was done (Figure S3a, b, c).

There are some similarities as small shiny particles and a rough look. However, the color of NaOH- and NaH-containing samples is different, being dark grey for the NaH-dominated Na-surface and brownish for the NaOH-dominated films under usage the same light source of the microscope for all pictures. This is an indicator that NaOH is not the only phase at the surface of H_2-treated sodium samples.

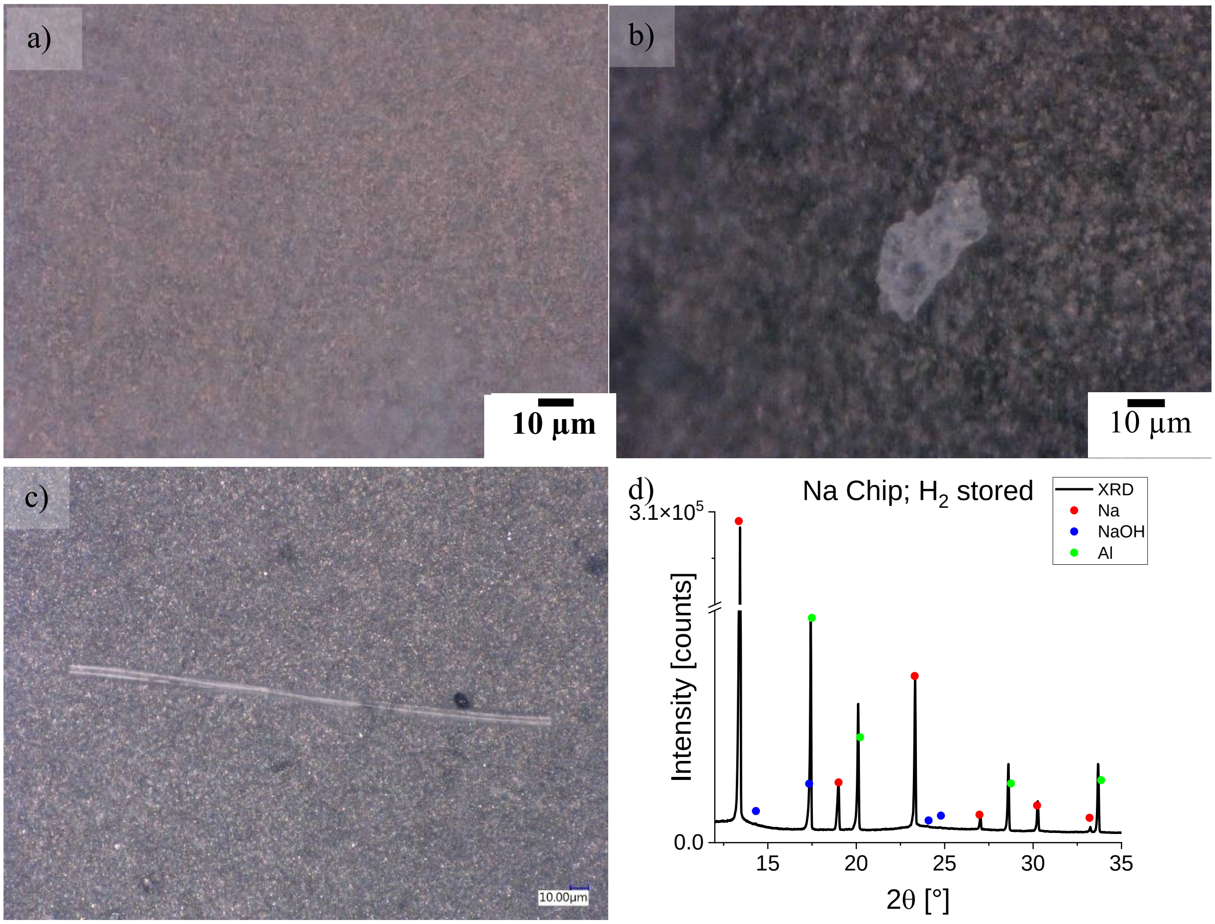

Figure S4. Comparison of freshly formed Na-surfaces with: a) NaOH-layer after storage of Na under ambient conditions for 5s, b) NaH-dominated layer after H_2-treatment. c) NaOH-dominated layer after H_2-treatment, d) XRD of H_2-treated Na with the NaH-dominated surface (Cu $K_{\alpha 1}$-radiation). No NaOH could be detected.

In the XRD results we could not detect any NaOH (Figure S4d). Therefore, formation of NaOH or NaH on the Na-surface depends on the surface impurities or Na chip pre-treatment, it is also possible that no NaOH forms at the surface of the metallic sodium.

5. Quantitative calibration of hydrogen evolution during TG-MS

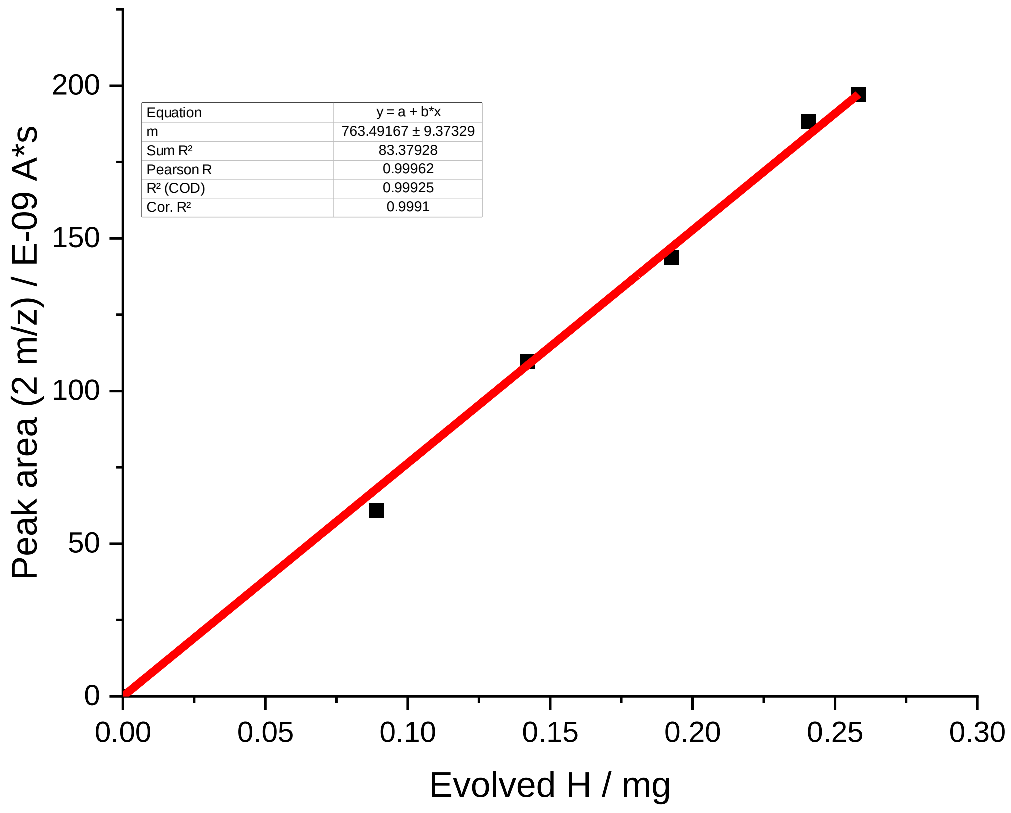

Figure S5. Calibration curve “ion current versus H-quantity” for determination of H-amount in H-containing materials.

6. Thermal behavior of sodium and NaOH

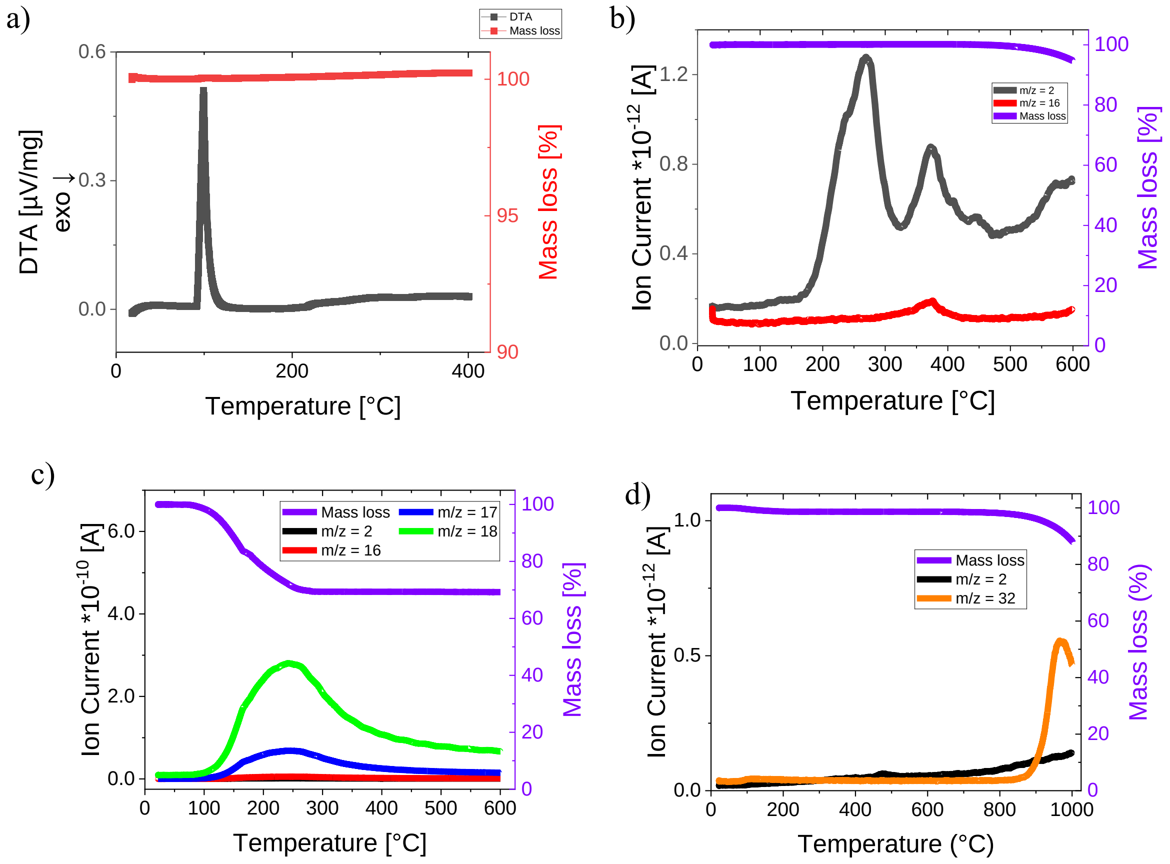

Figure S6. a) Melting behavior of sodium, b) thermal stability and mass-spectrometry analysis of metallic sodium up to 600°C. Thermal stability and mass spectrometry analysis of c) commercial water-containing NaOH up to 600°, showing only evaporation of water up to ca. 250 °C, and d) dried NaOH-sample. Its thermal decomposition starts above 850°C with evolution of O_2 and H_2 (the TG-curve is not mass-corrected).

7. Sodium deposition and stripping in case of H_2-exposed Na

Cycling of the H_2-treated sodium with the NaH-dominated surface in a half cell with pristine Na as a counter electrode shows a something lower potential development than a symmetric Na II Na cell, but both cells generally demonstrated a very similar Na-deposition and stripping behavior.

Further studies are in progress, with the focus to find a stable reference electrode for measurements such cells in a 3-electrode configuration.

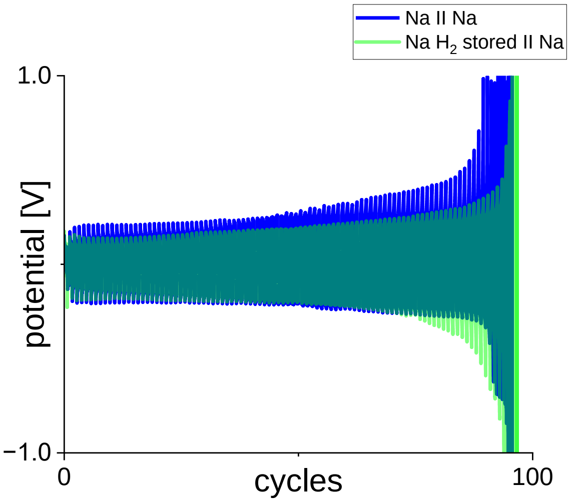

Figure S7. Comparison of deposition and stripping of a symmetric Na II Na cell with a Na H_2-treated II Na cell, cycled with 1 mA/cm² current density for 1h.

REFERENCES

(1) Lee, S.; Xu, H.; Xu, H.; Neuefeind, J. Crystal Structure of Moganite and Its Anisotropic Atomic Displacement Parameters Determined by Synchrotron X-Ray Diffraction and X-Ray/Neutron Pair Distribution Function Analyses. *Minerals* **2021**, *11* (3), 272. https://doi.org/10.3390/min11030272.
